# The complete mitochondrial genome of the green alga *Chloroidium sp. UTEX 3077* (Watanabea clade, Trebouxiophyceae)

**DOI:** 10.1080/23802359.2020.1711825

**Published:** 2020-01-16

**Authors:** Huanjun Zhang, Quanli Zhou, Yuanjin Liu, Wei Chen, Wei Sun, Haiyan Wang, Zhongquan Wang

**Affiliations:** Shandong Marine Resource and Environment Research Institute, Yantai, China

**Keywords:** *Chloroidium sp. UTEX 3077*, mitochondrial genome, phylogenetic analysis

## Abstract

The complete mitochondrial genome of *Chloroidium sp. UTEX 3077* was determined in this study. The circular genome was 90,774 bp in length with the GC content of 38.8%. It contained 30 protein-coding genes (PCGs), 22 transfer RNA (tRNA) genes and 2 ribosome RNA (rRNA) genes. A phylogenetic analysis based on the 8 mitochondrial genomes of Trebouxiophyceae indicated that *Chloroidium sp. UTEX 3077* grouped with Chlorellales.

Green algae are important ecological groups that play key roles as primary producers and are becoming as viable sources of commercial compounds in the fuel, food, and pharmaceutical industries (Hoek et al. 1995). Species of Chloroidium are widely distributed across different habits such as marine, terrestrial, and freshwater. They have been described under different specific and generic names according to their origin and morphology over the past century. They usually have high phenotypic plasticity, and therefore, easily adapt to different environments. These species are generally characterized by high phenotypic plasticity, ellipsoidal cell shape, unequal autospores during reproduction, small cell size, and parietal chloroplasts, as well as by the biochemical capability to synthesize and accumulate the rather unusual polyol, ribitol (Rindi and Guiry 2004; Karsten et al. 2005). These unique ecophysiological and morphological characteristics could explain the different distribution pattern compared to the other strains. Here, we aimed to assemble and characterize mitochondrial genome of *Chloroidium sp. UTEX 3077* to provide a better understanding of the evolution and genetics of Trebouxiophyceae.

The complete mitochondrial genome of *Chloroidium sp. UTEX 3077* was initially assembled from previously published Illumina sequencing data (SRR4434583), which was used as a PCR primer guide. The species was provided by the Ocean University of China in Qingdao (OUC-100302). The whole mitochondrial genome was sequenced with 90 pairs of primers and assembled using CAP3 software (Huang and Madan 1999). The assembled plastid genome was then annotated by DOGMA (Wyman et al. 2004) and was submitted to GenBank with accession numbers MN646686. The complete *Chloroidium sp. UTEX 3077* mitochondrial genome is 90,774 bp in length and contained 30 PCGs (*atp*1, *atp*9, *atp*8, *apt*4, *atp*6, *nad*7, *nad*5, *nad*2, *nad*6, *nad*4, *nad*1, *nad*3, *nad*9, *cox*1, *cox*2, *cox*3, *cob*, *ORF*148, *ORF*214, *Tat*C, *rps*12, *rps*7, *rps*19, *rps*4, *rps*13, *rps*14, *rps*3, *rpl*5, *rpl*16, *rpl*10), 22 tRNA genes, and 2 rRNA genes. The overall GC content is 38.8%.

A phylogenetic analysis was carried out with *Chloroidium sp. UTEX 3077* and 7 other complete mitochondrial genomes of species from the Trebouxiophyceae group. *Chlamydomonas reinhardtii* from Chlorophyceae was included as an outgroup. Eight concatenated protein-coding amino acid sequences were aligned using the program MAFFT (Katoh et al. 2005) and were trimmed using trimAl with the option ‘nogaps’ (Capella-Gutiérrez et al. 2009). Maximum likelihood (ML) analysis was conducted using RaxML with the JTT + G+I substitution model (Stamatakis 2014). The green alga *Chloroidium sp. UTEX 3077* in Watanabe clade clustered with Chlorellales with high bootstrap supported (Figure 1). The ML tree also indicated that the unclassified *Trebouxiophyceae sp. MX-AZ01* located in the clade of Trebouxiophyceae incertae sedis.

**Figure 1.**
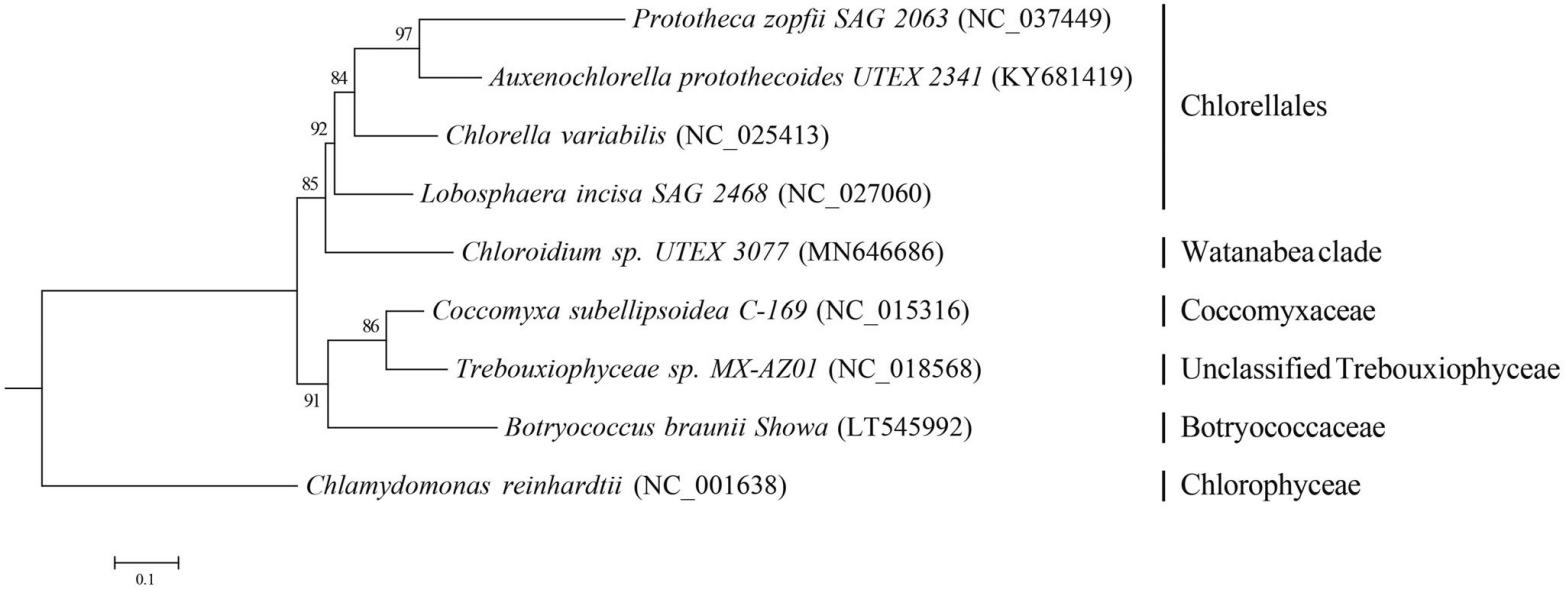
Maximum likelihood (ML) phylogenetic tree of the *Chloridium sp. UTEX 3077* and 8 other species based on the concatenated sequences of 8 protein-coding genes. Numbers on nodes indicate bootstrap support value, based on 1000 replicates the Genbank accession numbers were in brackets.
